# RNase L limits host and viral protein synthesis via inhibition of mRNA export

**DOI:** 10.1126/sciadv.abh2479

**Published:** 2021-06-04

**Authors:** James M. Burke, Alison R. Gilchrist, Sara L. Sawyer, Roy Parker

**Affiliations:** 1 Department of Biochemistry, University of Colorado Boulder, Boulder, CO 80303, USA.; 2 Department of Molecular, Cellular, and Developmental Biology, University of Colorado Boulder, Boulder, CO 80309, USA.; 3 Howard Hughes Medical Institute, University of Colorado Boulder, Boulder, CO 80303, USA.

## Abstract

RNase L is widely thought to limit viral protein synthesis by cleaving host rRNA and viral mRNA, resulting in translation arrest and viral mRNA degradation. Here, we show that the mRNAs of dengue virus and influenza A virus largely escape RNase L–mediated mRNA decay, and this permits viral protein production. However, activation of RNase L arrests nuclear mRNA export, which strongly inhibits influenza A virus protein synthesis and reduces cytokine production. The heterogeneous and temporal nature of the mRNA export block in individual cells permits sufficient production of antiviral cytokines from transcriptionally induced host mRNAs. This defines RNase L–mediated arrest of mRNA export as a key antiviral shutoff and cytokine regulatory pathway.

## Teaser

RNase L–mediated shutoff of nuclear mRNA export limits viral protein synthesis and regulates antiviral cytokine production.

## INTRODUCTION

Double-stranded RNA (dsRNA) is a viral-associated molecular pattern that initiates innate immune programs in mammalian cells (*1*, *2*). In response to dsRNA, cells induce the expression of antiviral proteins such as cytokines, which prime an antiviral state in infected and noninfected cells. Simultaneously, cells limit translation to reduce viral protein production. For decades, activation of the cytoplasmic endoribonuclease, RNase L, in response to viral dsRNA has been thought to limit viral protein synthesis by cleaving host ribosomal RNA (rRNA), resulting in arrest of translation (*3*–*7*). However, two recent studies indicate that RNase L–mediated cleavage of rRNA does not arrest translation. First, flaviviruses, including Zika virus and dengue virus (DENV), can replicate despite activating RNase L–mediated rRNA decay (*8*). Second, rapid and widespread decay of host mRNAs by RNase L accounts for RNase L–mediated reduction in translation (*9*, *10*). In light of these findings, it is unclear how RNase L limits viral protein synthesis, how some viruses escape the effects of RNase L, and how RNase L activation affects host antiviral protein production.

## RESULTS

### DENV mRNAs escape RNase L–mediated mRNA decay and produce protein

DENV is a flavivirus [+single-stranded RNA (+ssRNA) virus] that replicates in the cytoplasm. To investigate the mechanism by which DENV evades the effects of RNase L (*8*), we infected parental [wild-type (WT)] and RNase L–knockout (KO) (RL-KO) A549 cells with DENV serotype 2 and performed single-molecule fluorescence in situ hybridization (smFISH) for *DENV* mRNA and *GAPDH* (glyceraldehyde-3-phosphate dehydrogenase) mRNA, which is degraded when RNase L is active (*9*) and also stained for DENV NS3 protein production by immunofluorescence.

**Fig. 1:**
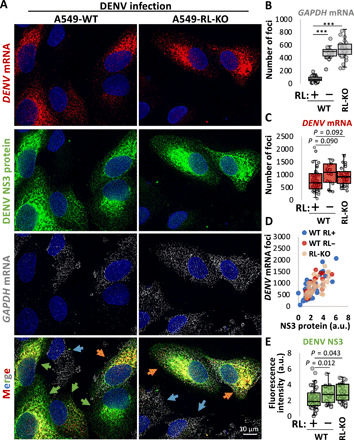
DENV mRNAs escape RNase L–mediated mRNA decay and are translated. (**A**) smFISH for *DENV* mRNA and *GAPDH* mRNA and immunofluorescence for DENV NS3 protein in WT and RL-KO A549 cells 48 hours after infection with DENV (MOI = 0.1). Images are from a single *Z* plane. Orange arrow: RNase L not activated (*GAPDH* mRNA not degraded); green arrow: RNase L activated (*GAPDH* mRNA degraded); blue arrow: uninfected cells (neither DENV mRNA nor NS3 detected). (**B**) Quantification of GAPDH mRNA in WT and RL-KO cells. WT cells in which *GAPDH* mRNA levels were lower than the lowest level observed in RL-KO cells were designated as RNase L active (RL+). WT cells with GAPDH mRNA levels above that threshold were designated RNase L inactive (RL–). (**C**) Quantification of *DENV* mRNA foci. (**D**) Scatterplot of DENV mRNA foci and DENV NS3 immunofluorescence. (**E**) Similar to (B) and (C) but quantifying the integrated density (a.u., arbitrary units) of DENV NS3 immunofluorescence as represented in (A).

DENV-infected cells frequently activated RNase L, as observed by a strong reduction of *GAPDH* mRNA in most DENV-infected WT but not RL-KO cells (Fig. 1, A and B, and fig. S1). DENV-infected cells that activated RNase L showed a small, but not statistically significant, reduction in DENV mRNA levels as compared to WT cells that failed to activate RNase L or RL-KO cells (Fig. 1C). Moreover, DENV protein production was strongly correlated with DENV mRNA levels (Fig. 1D) and only showed small decreases in cells with activated RNase L (Fig. 1E). Thus, DENV RNA is largely unaffected by RNase L–mediated mRNA decay. Consequently, this permits DENV protein production, consistent with cytoplasmic mRNAs having the capacity to be translated during the RNase L response (*9*, *10*).

### The host immune response inhibits mRNA export via RNase L activation

**Fig. 2:**
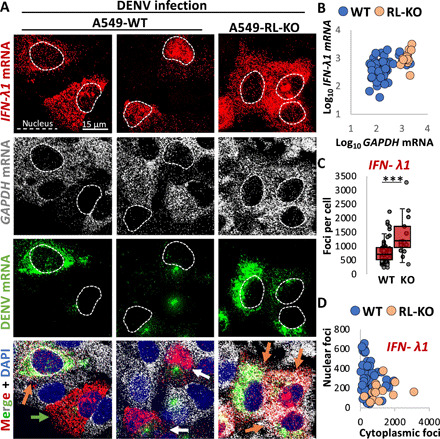
RNase L inhibits nuclear export of interferon (IFN) mRNAs in response to DENV infection. (**A**) smFISH for *IFN-*λ*1* mRNA, *GAPDH* mRNA, and *DENV* mRNA in WT and RL-KO A549 cells 48 hours after infection with DENV (MOI = 0.1). Orange arrow: DENV-positive but RNase L not activated (*GAPDH* mRNA not degraded); green arrow: DENV positive, RNase L activated (*GAPDH* mRNA degraded), and *IFN-*λ*1* mRNA localized to cytoplasm; white arrow: DENV positive, RNase L activated, and *IFN-*λ*1* mRNA retained in the nucleus. (**B**) Scatterplot of *GAPDH* mRNA and *IFN-*λ*1* mRNA in individual cells. (**C**) Quantification of smFISH foci in WT and RL-KO cells infected with DENV and that induced *IFN-*λ*1* mRNA expression. (**D**) Scatterplot of the number of smFISH foci of *IFN-*λ*1* mRNA localized to the nucleus or cytoplasm in individual WT and RL-KO cells as represented in (A).

Examination of host cytokine mRNAs induced by DENV infection in WT and RL-KO A549 cells revealed two important points. First, we observed that antiviral cytokine mRNAs largely escape RNase L–mediated mRNA decay. This is based on the observation that in some WT cells that activated RNase L–mediated mRNA decay (degraded GADPH mRNA), *IFN-*λ*1* (interferon-λ*1*) and *IFN-*β mRNAs were abundant in the cytoplasm, often at levels comparable to those observed in RL-KO cells (Fig. 2, A and B, and figs. S2, A and B, and S3, A and B). Moreover, while median GAPDH mRNA levels were reduced more than 10-fold by RNase L (Fig. 1B), median *IFN* mRNA levels were only reduced 2-fold by RNase L (Fig. 2C and fig. S3C). Thus, cytokine mRNAs that reach the cytoplasm largely escape RNase L–mediated mRNA decay during DENV infection, consistent with our observations during the dsRNA response (*9*).

**Fig. 3:**
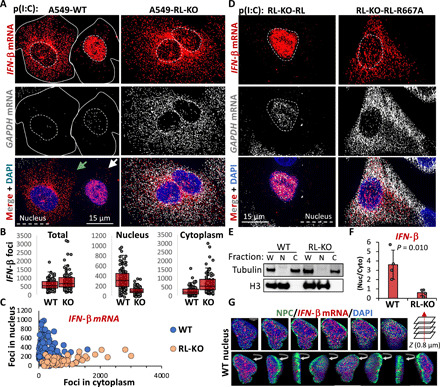
RNase L inhibits nuclear export of IFN mRNAs in response to dsRNA. (**A**) smFISH for *IFN-*β and *GAPDH* mRNAs in WT and RL-KO A549 cells 8 hours after poly(I:C) lipofection. Solid lines mark the cell boundary of WT cells since it is difficult to view cell boundaries with nuclear-retained IFN mRNA. (**B**) Quantification of *IFN-*β smFISH foci, as represented in (A), in individual cells (dots). (**C**) Scatterplot of the nuclear and cytoplasmic *IFN-*β foci in individual WT and RL-KO cells, as represented in (A). (**D**) smFISH for *IFN-*β and *GAPDH* mRNAs in RL-KO cells expressing RNase L or RNase L-R667A catalytic mutant via lentiviral transduction after poly(I:C) lipofection. (**E**) Immunoblot of whole cell (W), nuclear (N), and cytoplasmic (C) fractions from WT and RL-KO cells 6 hours after poly(I:C) used for RT-qPCR. (**F**) qRT-PCR quantification of *IFN-*β mRNA from nuclear-cytoplasmic fractions as shown in (E). Bars represent the average 2^−ΔCt^ (nuclear-cytoplasm) ± SD (*n* = 4). Dots represent independent experiments. The *P* value was determined using Student’s *t* test. (**G**) Immunofluorescence for NPC and smFISH for *IFN-*β mRNA in a WT cell displaying nuclear retention of *IFN-*β mRNA.

Unexpectedly, we also observed accumulation of *IFN*-β and *IFN-*λ*1* mRNAs within the nucleus of some DENV-infected WT cells (Fig. 2, A to D, and figs. S2, A and B, and S3, A to D). In principle, this mRNA export block could be mediated by DENV or could be a consequence of the cellular response to dsRNA. To distinguish these possibilities, we transfected cells with polyinosinic:polycytidylic acid [poly(I:C)], a viral dsRNA mimic, and examined whether mRNA export was affected. We observed that poly(I:C) transfection was sufficient to activate RNase L, to induce *IFN* mRNAs, and to block mRNA export in a fraction of WT cells (Fig. 3, A to C, and fig. S4, A to C). This demonstrates a previously unidentified aspect of the host innate immune response that blocks nuclear mRNA export.

Several observations demonstrate that this block to mRNA export requires activation of RNase L–mediated mRNA degradation. First, nuclear retention of *IFN* mRNAs was only observed in DENV-infected WT cells that activated RNase L–mediated decay of *GAPDH* mRNA (Fig. 2, A to D). In contrast, neither WT cells that failed to activate RNase L (as assessed by the lack of *GAPDH* mRNA degradation) nor RL-KO cells that induced cytokine mRNAs displayed the mRNA export block (Fig. 2, A to D). Second, the mRNA export block in response to poly(I:C) was also RNase L–dependent, as we did not observe *IFN* mRNA accumulation in the nucleus of RL-KO cells (Fig. 3, A to C, and fig. S4, A to C). Third, the block to mRNA export requires RNase L catalytic activity since rescuing the RL-KO cells with RNase L, but not RNase L-R667A (a catalytic mutant), restored nuclear retention of *IFN-*β mRNA in response to poly(I:C) (Fig. 3D). Nuclear accumulation of IFN-β mRNAs was also observed by quantitative reverse transcription polymerase chain reaction (RT-qPCR) of nuclear and cytoplasmic biochemical fractions (Fig. 3, E and F). Thus, activation of RNase L is sufficient to induce a block to nuclear mRNA export.

Immunofluorescence assay showed that nuclear-retained *IFN-*β mRNA is internal to the nuclear pore complex (NPC) and not retained at the site of transcription (Fig. 3G), which can be observed with many RNA processing defects (*11*). The block to nuclear export is independent of PKR (protein kinase RNA-activated), since nuclear accumulation of mRNAs after poly(I:C) treatment is also observed in PKR-KO cells (fig. S4D). The nuclear block appears independent of RNase L–mediated apoptosis since the Z-VAD-FMK caspase inhibitor did not inhibit it (fig. S4E).

Our observations indicate that RNase L activation inhibits the chromosomal region maintenance 1 (CRM-1) mRNA export pathway, which is a specialized mRNA export pathway used by mRNAs without introns and/or that contain AU-rich elements (AREs) (*12*). The *IFN-*β mRNA, which lacks introns and contains AREs, is thought to exit the nucleus by the CRM-1 mRNA export pathway (*13*). Treatment of RL-KO cells with the CRM-1 inhibitor, leptomycin B, phenocopied the RNase L–dependent nuclear retention of *IFN-*β mRNA observed in WT cells (fig. S4F). Thus, nuclear accumulation of *IFN-*β mRNA is a result of RNase L–dependent inhibition of CRM-1–mediated mRNA export.

RNase L activation also inhibits the NXF1-TREX (nuclear RNA export factor 1-transcription export complex) bulk mRNA export pathway that acts on spliced mRNAs since *IFN-*λ*1* mRNA contains introns and is insensitive to leptomycin B (fig. S4G). Moreover, the *GAPDH* mRNA, which is also spliced and presumably exported by the NFX-TREX pathway, also shows nuclear retention in cells with activated RNase L (Fig. 3D). These observations argue that RNase L activation inhibits both the CRM-1 and the NFX1-TREX mRNA export pathways.

### Activation of RNase L inhibits influenza virus mRNA export and protein synthesis

We hypothesized that RNase L–mediated inhibition of mRNA export may be important for limiting viral protein synthesis for viruses that must export their mRNAs from the nucleus. For example, influenza (–ssRNA virus) replicates in the nucleus and must export its mRNAs to the cytosol and is inhibited 100- to 1000-fold by RNase L (*14*). To test whether RNase L activation blocked influenza mRNA export, we infected WT and RL-KO A549 cells with influenza A/Udorn/72 virus (IAV). Since the IAV NS1 protein (non-structural protein 1) strongly attenuates RNase L activation (fig. S5A) (*14*), we transfected cells with poly(I:C) 1 hour after infection to promote RNase L activation. We performed smFISH 7 hours after infection, when IAV output is in log phase growth and when RNase L reduces viral output by greater than 100-fold (*14*).

**Fig. 4:**
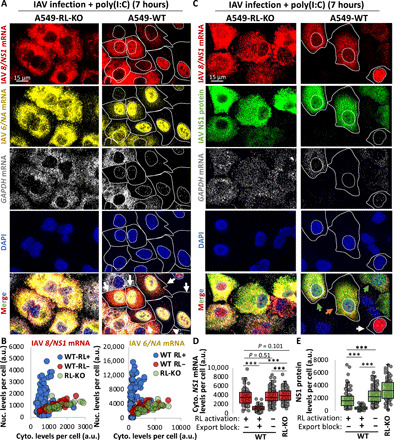
RNase L–mediated inhibition of mRNA export inhibits influenza virus protein synthesis. (**A**) smFISH for IAV *NA* and *NS1* mRNAs 7 hours after infection (MOI = 0.5). Cells were transfected with poly(I:C) 1 hour after infection. *GAPDH* mRNA degradation is a marker for RNase L activation. Additional images are shown in fig. S5 (B and C). White arrows, nuclear retention of *NA* and *NS1*; blue arrows, nuclear retention of *NA* but not *NS1*. (**B**) Scatterplot of nuclear intensity and cytoplasmic intensity of *NA* or *NS1* mRNAs from individual WT cells with active RNase L (RL+; loss of *GAPDH* mRNA) or inactive RNase L (RL−; contain *GAPDH* mRNA) and RL-KO cells. (**C**) Similar to (A) but smFISH for *NS1* mRNA and *GAPDH* mRNA and immunofluorescence staining of NS1 protein. Green arrow: RL−, cytoplasmic *NS1* mRNA+; orange arrow: RL+, cytoplasmic *NS1* mRNA+; white arrow: RL+, nuclear *NS1* mRNA retention+. (**D**) Cytoplasmic staining intensity of *NS1* mRNA in RL− WT cells, RL+ WT cells displaying either *NS1* mRNA nuclear retention (export block+) or cytoplasmic localization (export block−), and RL-KO cells. WT cells with export block were defined as having a nuclear-to-cytoplasmic *8/NS1 mRNA* intensity ratio that was higher than the maximum ratio observed in RL-KO cells for each replicate (fig. S6C). (**E**) Similar to (D) but plotting immunofluorescent intensity of NS1 protein. Statistical significance (**P < 0.005) was determined by *t* test analysis.

An important result was that most WT cells that activated RNase L (as assessed by degradation of the *GAPDH* mRNA) limited the export of IAV *NA* (neuraminidase) and *NS1* mRNAs (Fig. 4, A and B, and fig. S5, B to E). Nuclear retention of these mRNAs did not occur in WT cells that failed to activate RNase L–mediated mRNA decay or in RL-KO cells. This demonstrates that RNase L activation leads to the inhibition of IAV mRNA export.

We observed that in cells that activated RNase L–mediated mRNA decay but failed to trigger the mRNA export block, influenza mRNAs were abundant in the cytoplasm (Fig. 4, A to C, and figs. S5, B to F, and S6A), at levels comparable to those observed in WT cells that did not activate RNase L and RL-KO cells (Fig. 4D and figs. S5, B to F). This argues that IAV mRNAs escape RNase L–mediated mRNA decay and could potentially be translated for protein production, similar to DENV mRNAs (Fig. 1). This suggested that the RNase L–mediated mRNA export block was the mechanism by which RNase L limits influenza protein synthesis.

To test this hypothesis, we examined the relationship between influenza NS1 protein production and the *NS1* mRNA export block in individual cells (Fig. 4C and fig. S6, A to C). We observed that cells displaying RNase L activation (*GADPH* mRNA degradation), but successful *NS1* mRNA export, produced NS1 protein at levels comparable, albeit slightly less, to WT cells that did not activate RNase L and RL-KO cells (Fig. 4, C to E). In contrast, WT cells in which *NS1* mRNA export was inhibited by RNase L activation displayed significantly less NS1 protein production (Fig. 4, C to E). Last, cytoplasmic levels of *NS1* mRNA strongly correlated with NS1 protein levels with or without RNase L activation (fig. S6B). These observations demonstrate that RNase L–mediated inhibition of mRNA export is a primary mechanism by which RNase L reduces influenza protein production.

### RNase L–mediated inhibition of mRNA export limits antiviral protein production

RNase L–mediated inhibition of mRNA export would also be expected to limit translation of transcriptionally induced antiviral mRNAs, which could be detrimental to the antiviral response. However, since this response was heterogeneous with respect to individual cells (Figs. 2 and 3), we suspected that the RNase L–mediated mRNA export block would reduce, but not abolish, antiviral cytokine production. Such a function would be important for ensuring cytokine production while potentially preventing the overproduction of cytokines, which can cause cytokine storm phenomena.

**Fig. 5:**
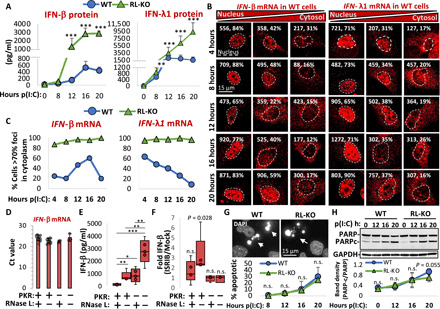
RNase L–mediated mRNA export block contributes to inhibition of antiviral cytokine production. (**A**) ELISAs for IFN-β and IFN-λ1. Markers represent the mean SD from at least six replicates. Statistical significance (*P < 0.05; **P < 0.005; ***P < 0.0005) was determined by *t* test analysis. (**B**) WT A549 cells displaying varying degrees of nuclear retention of *IFN-*β or *IFN-*λ*1* mRNA after poly(I:C). The number and percentage of smFISH foci localized to the nucleus are shown in the top right corners. Nuclei were determined by DAPI staining (not shown for space and clarity) and are outlined. Additional images are shown in figs. S8 and S9. (**C**) The percentage of cells displaying 70% or greater *IFN-*β or *IFN-*λ*1* smFISH foci in the cytoplasm derived from fig. S9D. (**D**) RT-qPCR for *IFN-*β mRNA in WT, RL-KO, PKR-KO, and RL/PKR-dKO cell lines 6 hours after poly(I:C). Bars represent the average cycle threshold (Ct) ± SD. Each dot represents a Ct value from an individual experiment. (**E**) Quantification of IFN-β via ELISA 12 hours after poly(I:C). (**F**) Fold change in IFN-β secretion via ELISA 12 hours after poly(I:C) with or without cotreatment with ISRIB. (**G**) WT and RL-KO cells stained with DAPI to visualize apoptotic cells (white arrows). The graph below shows the mean percentage and SD of apoptotic cells. (**H**) Immunoblot analysis of PARP and caspase-mediated cleavage fragment of PARP (PARPc). The graph below shows the mean ± SD of PARPc/PARP ratio. n.s., not significant.

Three primary observations support these hypotheses. First, RNase L expression reduced, but did not abolish, secretion of IFN-β and IFN-λ1 proteins in a manner dependent on its catalytic activity in response to poly(I:C) (Fig. 5A and fig. S7), consistent with a previous study (*15*).

Second, nuclear retention of *IFN-*β and *IFN-*λ*1* mRNAs was differential with respect to time (Fig. 5, B and C, and figs. S8, A to D, and S9, A to D), and this correlated with RNase L–dependent repression of protein production (Fig. 5A). Specifically, ~75% of WT cells displayed nuclear retention of *IFN-*β mRNA before 12 hours post-poly(I:C) lipofection (Fig. 5C) when RNase L inhibited IFN-β levels by 10-fold (Fig. 5A). At 12 and 16 hours after poly(I:C), cytoplasmic *IFN-*β mRNA levels increased in WT cells (Fig. 5C), which preceded/coincided with an increase in IFN-β (Fig. 5A). In contrast, fewer cells (~35%) displayed nuclear retention of *IFN-*λ*1* mRNA at early times after poly(I:C) (Fig. 5C), and this correlated with comparable IFN-λ1 levels from WT and RL-KO cells at 8 and 12 hours after poly(I:C) (Fig. 5A). However, localization of the *IFN-*λ*1* mRNA progressively increased to nucleus over time in WT cells (Fig. 5C), correlating with a cessation of IFN-λ1 secretion at late times after poly(I:C) (Fig. 5A). These data show that RNase L–mediated inhibition of *IFN* mRNA nuclear export correlates with reduction in IFN protein production.

Third, several observations indicate that RNase L inhibits IFN-β protein production at 12 hours after poly(I:C), specifically via the export block as opposed to mRNA decay. The twofold reduction of *IFN-*β mRNA by RNase L before 12 hours post-poly(I:C) lipofection is insufficient to account for the 10-fold reduction of IFN-β protein observed at 12 hours after poly(I:C) (Fig. 5, A to D, and fig. S9C). Moreover, despite an equivalent ~2-fold reduction in *IFN-*λ*1* and *IFN-*β mRNAs by RNase L during this time, IFN-λ1 protein levels were largely unaffected by RNase L (Fig. 5A and fig. S9, C and D), correlating with *IFN-*λ*1* mRNA being predominantly cytoplasmic, whereas *IFN-*β mRNA was primarily nuclear at early times after poly(I:C) (Fig. 5C). These data argue that nuclear retention, as opposed to RNA decay, of the *IFN-*β mRNA accounts for reduced IFN-β protein production.

We also considered the possibility that RNase L limits IFN-β protein production by activating eukaryotic initiation factor–2α (eIF2α) kinases (*9*), particularly PKR (*16*), which would promote phosphorylation of eIF2α (p-eIF2α) and repress translation. However, the cumulative increase of IFN-β production in RNase L/PKR double KO cells in comparison to PKR-KO or RL-KO cells argues that RNase L inhibits IFN-β production independently of PKR (Fig. 5E). We note that neither PKR nor RNase L markedly affected *IFN-*β mRNA levels (Fig. 5D). Moreover, integrated stress response inhibitor (ISRIB) treatment, which counteracts the effects of p-eIF2α (*17*), failed to increase IFN-β in cells that express RNase L (Fig. 5F). Thus, RNase L reduces translation of *IFN-*β mRNA independently of p-eIF2α.

Last, RNase L–mediated apoptosis (*18*, *19*) is not responsible for limiting IFN-β production at 12 hours after poly(I:C), since we did not observe a notable number of apoptotic cells or caspase-mediated poly[adenosine 5′-diphosphate (ADP)–ribose] polymerase (PARP) cleavage before 12 hours after poly(I:C) (Fig. 5, G and H), consistent with live-cell imaging studies (*20*). Moreover, we did not observe a substantial difference in the rate of cells initiating apoptosis or in the rate of caspase-mediated PARP cleavage between WT and RL-KO cells (Fig. 5, G and H).

Together, these observations argue that global reductions in translation via mRNA decay, translation arrest, or apoptosis do not account for RNase L–mediated inhibition of IFN-β. Instead, our data demonstrate that RNase L reduces type I and type III IFN production by blocking the export of their mRNAs to the cytoplasm.

## DISCUSSION

We present several observations documenting new aspects of the innate immune response. First, we show that cytoplasmic DENV and IAV mRNAs escape RNase L–mediated decay and, consequently, produce protein during the RNase L response (Figs. 1 and 4). These data indicate that RNase L activation does not arrest translation via rRNA cleavage, consistent with recent studies (*9*, *10**)*, and elucidate how some viruses, such as ZIKA virus, can synthesize proteins despite robust RNase L–mediated rRNA decay (*8*). Similar to host antiviral mRNAs (*9*, *10*), the high transcriptional rates of viral mRNAs and their structure, as well as their localization to replication factories and association with viral proteins (*8*), likely contribute to their ability to escape the effects of RNase L–mediated mRNA decay.

We also identified a new mechanism by which RNase L reduces host and viral gene expression, whereby RNase L activation inhibits mRNA export of host and viral mRNAs, which reduces their translation by preventing their association with ribosomes in the cytoplasm. This is based on the observations that host and viral mRNAs accumulate in the nucleus of cells that have activated RNase L, and this correlates to reduced protein expression from these transcripts (Figs. 2 to 5).

The mechanism by which activation of RNase L inhibits mRNA export remains to be discovered. Several observations are consistent with RNA export being inhibited following widespread cytoplasmic RNA decay due to an influx of cytoplasmic RNA binding proteins (RBPs), which then limit the interaction of RNA export factors with nuclear mRNAs. For example, in addition to RNase L–mediated mRNA decay, widespread degradation of host cytoplasmic mRNAs by the SOX and VHS nucleases encoded by Kaposi’s sarcoma–associated herpesvirus and herpes simplex virus 1, respectively, leads to the import of cytosolic RBPs into the nucleus in conjunction with mRNA export inhibition (*9*, *20*–*24*). Further supporting this model, RNase L–mediated inhibition of mRNA export is dependent on its catalytic activity and occurs after mRNA decay (Fig. 3, A to D). Elucidating the mechanism by which RNase L–mediated mRNA decay inhibits mRNA export will be a key focus of future studies.

One function of RNase L–mediated inhibition of mRNA export is to limit viral protein production by limiting gene expression from viruses that replicate in the nucleus. Our data suggest that this is a primary mechanism by which RNase L limits influenza protein production since nuclear accumulation of influenza mRNAs is required for a reduction in their translation in cells with activated RNase L (Fig. 4). Since most DNA viruses must export their mRNAs from the nucleus and can activate RNase L (*25*–*28*), this could be a broad antiviral mechanism.

A second function of RNase L–mediated inhibition of mRNA export is to regulate cytokine production. We observed that the reduction of IFN-β protein due to RNase L correlates with the mRNA export block and cannot be explained by RNase L–mediated global reduction in translation, mRNA degradation, or enhanced apoptosis (Fig. 5). Thus, RNase L activation limits cytokine production by trapping cytokine mRNAs in the nucleus in a fraction of the cells (Fig. 5). We suggest that the heterogeneous and temporal nature between individual cells allows for sufficient production of cytokines to limit viral replication (Figs. 3, A and B, and 5, A to C, and figs. S7 to S9) (*29*). Moreover, our data indicate that type III IFNs are less repressed by RNase L than type I IFNs at early times after dsRNA (Fig. 5, A to C, and figs. S7 to S9). This would promote localized epithelial cell–specific antiviral signaling before systemic type I IFN signaling (*30*). Combined, these functions may prevent systematic overproduction of cytokines, which can lead to cytokine storm, sepsis, and autoimmune disorders in response to viral infection (*31*–*33*).

## MATERIALS AND METHODS

### Cell culture

The A549 cell line was provided by C. Sullivan (The University of Texas at Austin). The A549 RL-KO cell line and RNase L and RNase L-R667A RL-KO cell lines are described in (*9*). The generation of PKR-KO and RL/PKR-KO and lines are described in (*20*). Cells were maintained at 5% CO_2_ and 37°C in Dulbecco’s modified Eagle’s medium supplemented with fetal bovine serum (10% v/v) and penicillin-streptomycin (1% v/v). Cells tested negative for mycoplasma contamination by the BioFrontiers Cell Culture Core Facility. Cells were transfected with poly(I:C) HMW (high molecular weight) (InvivoGen: tlrl-pic) using 3 μl of Lipofectamine 2000 (Thermo Fisher Scientific) per 1 μg of poly(I:C). Cells were treated with caspase inhibitor Z-VAD-FMK (Promega: G7231) at 20 μM concentration.

### Virus infections

A549 cells were infected with DENV serotype 2 16681 strain at a multiplicity of infection (MOI) of 0.1. Cells were fixed 48 hours after infection. Cells were infected with IAV strain at an MOI of 0.5. Cells were fixed 7 hours after infection.

### Immunoblot analyses

Immunoblot analysis was performed as described in (*9*). Rabbit anti-GAPDH (Cell Signaling Technology: 2118L) was used at 1:2000. Anti-rabbit immunoglobulin G (IgG), horseradish peroxidase (HRP)–linked antibody (Cell Signaling Technology: 7074S) was used at 1:3000. Anti-mouse IgG, HRP-linked antibody (Cell Signaling Technology: 7076S) was used at 1:10,000. Rabbit anti-PARP (Cell Signaling Technology: 9452S) was used at 1:1500. Histone H3 antibody (Thermo Fisher Scientific; NB500-171) was used at 1:1000.

### Immunofluorescence and smFISH

smFISH was performed following the manufacturer’s protocol (https://biosearchassets.blob.core.windows.net/assets/bti_custom_stellaris_immunofluorescence_seq_protocol.pdf). GAPDH smFISH probes labeled with Quasar 570 dye (SMF-2026-1) or Quasar 670 dye (SMF-2019-1) were purchased from Stellaris. Custom IFNB1, IFNL1, IAV, and DENV smFISH probes were designed using Stellaris smFISH probe designer (Biosearch Technologies) available online at http://biosearchtech.com/stellaris-designer. Reverse complement DNA oligos were purchased from IDT (data file S1). The probes were labeled with ATTO-633 using ddUTP-Atto633 (Axxora: JBS-NU-1619-633), ATTO-550 using 5-Propargylamino-ddUTP (Axxora; JBS-NU-1619-550), or ATTO-488 using 5-Propargylamino-ddUTP (Axxora; JBS-NU-1619-488) with terminal deoxynucleotidyl transferase (Thermo Fisher Scientific: EP0161) as described in (*34*).

For immunofluorescence detection of IAV NS1, cells were incubated with the anti–influenza A virus NS1 rabbit antibody (GeneTex; GTX125990) at 1:1000 for 2 hours, washed three times, and then incubated with the goat anti-rabbit IgG H&L (heavy and light) fluorescein isothiocyanate (FITC) (Abcam; ab6717) at 1:2000 for 1 hour. After three washes, cells were fixed, and then smFISH protocol was performed. For detection of DENV NS3, cells were incubated with anti-DENV NS3 protein antibody (GeneTex: GTX124252) at 1:1000 for 2 hours and then incubated with goat anti-rabbit IgG H&L (Alexa Fluor 647) (Abcam: ab150079) for 1 hour. Mouse anti-NPC protein antibody (Abcam; ab24609) was used at 1:500 and detected with goat anti-mouse IgG H&L (FITC) (Abcam; ab97022) used at 1:2000.

### Microscopy and image analysis

Coverslips were mounted on slides with VECTASHIELD Antifade Mounting Medium with 4′,6-diamidino-2-phenylindole (DAPI) (Vector Laboratories; H-1200). Images were obtained using a wide-field DeltaVision Elite microscope with a 100× objective using a PCO Edge sCMOS camera. Between 10 and 15 *Z* planes at 0.2 μm per section were taken for each image. Deconvoluted images were processed using ImageJ with FIJI plugin. *Z* planes were stacked, and minimum and maximum display values were set in ImageJ for each channel to properly view fluorescence. Fluorescence intensity was measured in ImageJ. Single cells were outlined by determining the cell boundaries via background fluorescence and mean intensity, and integrated intensity was measured in the relevant channels. Imaris Image Analysis Software (Bitplane) (University of Colorado Boulder, BioFrontiers Advanced Light Microscopy Core) was used to quantify smFISH foci in nucleus and cytoplasm. Single cells were isolated for analysis by defining their borders via background fluorescence. Total foci above background threshold intensity were counted. Afterward, the nucleus marked with DAPI was masked, and foci were counted in the cell at the same intensity threshold cutoff, yielding the cytoplasmic foci count, from which the nuclear foci number could be determined.

### RT-qPCR

RT-qPCR for IFN-B1 was performed as described in (*9*). WT and RL-KO A549 cells (12-well format; 60% confluent) were transfected with or without poly(I:C). Six hours after transfection, crude nuclear and cytosolic fractions were obtained as described in (*35*). RNA was extracted from each fraction, treated with deoxyribonuclease (DNase) I (NEB) for 15 min, repurified via ethanol (75%) sodium acetate (0.3 M) precipitation, and resuspended in 15 μl of water. Equal volumes of RNA from each fraction were then reverse-transcribed using SuperScript III reverse transcriptase (Thermo Fisher Scientific) and polydT_(20)_ primer (Integrated DNA Technologies). cDNA was diluted to 100 μl. cDNA (2 μl) was added to qPCR reaction containing iQ SYBR green master mix (Bio-Rad) and 10 pmol of gene-specific primers. Reactions were run in duplicate or triplicate (technical replicates) on a CFX96 qPCR machine (Bio-Rad) using the standard two-step cycle.

### Quantification of secreted cytokines

WT and RL-KO A549 cells (six-well format, 1 ml or medium, and 70% confluency) were transfected with poly(I:C). At 6 and 12 hours after poly(I:C), 50 μl of medium was removed from the well and immediately assayed via enzyme-linked immunosorbent assay (ELISA) following the manufacturer’s instructions. IFN beta human ELISA Kit (Thermo Fisher Scientific; 414101) was used to quantify IFNB1. Human IL-29/IFN-lambda 1 ELISA Kit (Novus Biologicals; NBP1-84819) was used to quantify IFNL1. Time zero was taken by removing 50 μl of medium before poly(I:C) transfection.

## SUPPLEMENTARY MATERIALS

Supplementary material for this article is available at http://advances.sciencemag.org/cgi/content/full/7/23/eabh2479/DC1
